# The algorithmic management paradox: a structured integrative review of perceived autonomy on performance in digital workplaces

**DOI:** 10.3389/fpsyg.2026.1829522

**Published:** 2026-08-11

**Authors:** Feng Lu

**Affiliations:** Faculty of Business and Law, Deakin University, Melbourne, VIC, Australia

**Keywords:** algorithmic management, digital workplace, employee performance, integrative review, perceived autonomy, workplace surveillance

## Abstract

Digital workplaces increasingly delegate managerial functions such as task allocation, scheduling, monitoring, evaluation, feedback, and sanctioning to software systems. These systems can improve coordination speed, scalability, and consistency, but they also redistribute worker discretion in uneven ways. This article examines that tension as an algorithmic management autonomy paradox. Rather than asking whether algorithmic management is simply empowering or controlling, the review asks which facet of autonomy is affected, through which psychological mechanism, and with what consequences for different types of performance. A structured integrative review was conducted across Scopus, Web of Science, PsycINFO, ABI/INFORM, and the ACM Digital Library, supported by backward and forward citation tracing. The core search covered 2015 to January 2026 and used search blocks related to algorithmic management, digital monitoring, platform work, autonomy, fairness, stress, empowerment, and performance. The screening process identified 358 records, retained 276 after de-duplication, assessed 94 full texts, and produced a final corpus of 41 sources. The synthesis used a thematic coding matrix to classify algorithmic practices, autonomy facets, psychological mediators, outcome categories, moderators, evidence type, and level of analysis. The review makes a deliberately bounded contribution. It does not claim to propose a wholly new theory of algorithmic management; instead, it develops a middle-range framework that connects five groups of algorithmic practices with three autonomy facets, key psychological mechanisms, and differentiated performance outcomes. The synthesis suggests that algorithmic management is most likely to support sustainable performance when workers retain meaningful method and decision-making autonomy, when monitoring is developmental rather than punitive, and when transparency is actionable through explanation, contestability, and human review. Six propositions and a practical governance agenda are offered for future research and organizational design.

## Introduction

Managerial algorithms involve utilizing software, data analysis, and even artificial intelligence to perform managerial tasks, which human supervisors were traditionally expected to perform, such as task assignments, behavior monitoring, performance assessment, and behavioral enforcement (Lee et al., 2015; Jarrahi et al., 2021; Meijerink and Bondarouk, 2023). Though the concept appeared most on digital labor platforms, algorithmic management now permeates far further beyond gig work into warehouses, logistics, call centers, remote office settings, and professional knowledge work (Kellogg et al., 2020; Benlian et al., 2022; Milanez et al., 2025).

The central problem addressed in this article is not simply whether workers are controlled by algorithmic management. Many are. The more precise question is how algorithmic systems reshape perceived autonomy and when that reshaping supports or undermines performance. Digital workplaces often combine two apparently contradictory tendencies. On the one hand, algorithmic systems can increase flexibility by allowing workers to choose when to log in, where to work, or which opportunities to accept. On the other hand, the same systems can narrow discretion through continuous monitoring, opaque metrics, behavioral nudges, automated sanctions, and limited appeal options (Rosenblat and Stark, 2016; Wood et al., 2019; Cameron, 2024).

This article treats this tension as an autonomy paradox, but it defines the contribution cautiously. The paradox itself is not entirely new; platform work and surveillance studies have repeatedly shown that nominal independence can coexist with strong digital control. The contribution of this article is instead to specify the paradox more analytically. It explains why contradictory findings appear across the literature by distinguishing among autonomy facets, psychological mediators, outcome categories, and contextual boundary conditions.

Three clarifications structure the review. First, autonomy is not treated as a general feeling of freedom. The analysis distinguishes among scheduling autonomy, method autonomy, and decision-making autonomy. Second, performance is not treated as a single outcome. The review separates psychological states, behavioral performance outcomes, and sustainability outcomes. Third, algorithmic management is not equated with surveillance alone. It is examined as a bundle of software-mediated practices that include allocation and nudging, method standardization, surveillance and feedback, rewards and sanctions, and transparency and voice.

This framing shifts the discussion away from the broad claim that algorithmic management is either empowering or oppressive. A system that increases scheduling flexibility while reducing method discretion may affect engagement, efficiency, fatigue, and adaptive performance in different directions. Similarly, a monitoring system framed as developmental feedback may be experienced differently from one framed as punitive surveillance (Schlund and Zitek, 2024). The purpose of the review is therefore to build a structured synthesis that can explain these mixed effects and identify the conditions under which algorithmic management can support rather than deplete sustainable performance.

### Review design and scope

This article is explicitly positioned as a structured integrative review. An integrative review is appropriate because the focal problem spans organizational behavior, work design, labor studies, human resource management, information systems, human-computer interaction, and digital work research. The aim is not only to catalogue findings but also to integrate heterogeneous evidence into a conceptual framework that can guide future empirical research (Whittemore and Knafl, 2005; Torraco, 2005; Snyder, 2019).

### Review questions

The review was organized around three questions: (1) How do algorithmic management practices expand or constrain different facets of perceived autonomy? (2) Through which psychological mechanisms do these autonomy effects influence performance-related outcomes? (3) Which contextual conditions explain why similar algorithmic practices produce different effects across work settings?

### Search strategy and screening process

The literature search was conducted iteratively in Scopus, Web of Science, PsycINFO, ABI/INFORM, and the ACM Digital Library. The core search period was 2015 to January 2026, corresponding to the period in which algorithmic management became a distinct research topic. Earlier seminal work on job design, self-determination, empowerment, paradox theory, and performance was added through backward citation tracing when it was conceptually necessary.

Search strings combined three blocks: the managerial system, the psychological construct, and the outcome. Examples included: “algorithmic management” OR “algorithmic control” OR “management by algorithm” OR “digital monitoring” OR “electronic performance monitoring” OR “platform work” AND “autonomy” OR “job autonomy” OR “perceived autonomy” OR “empowerment” OR “fairness” OR “stress” AND “performance” OR “work engagement” OR “task performance” OR “quality” OR “adaptive performance” OR “turnover intention.”

**Figure 1:**
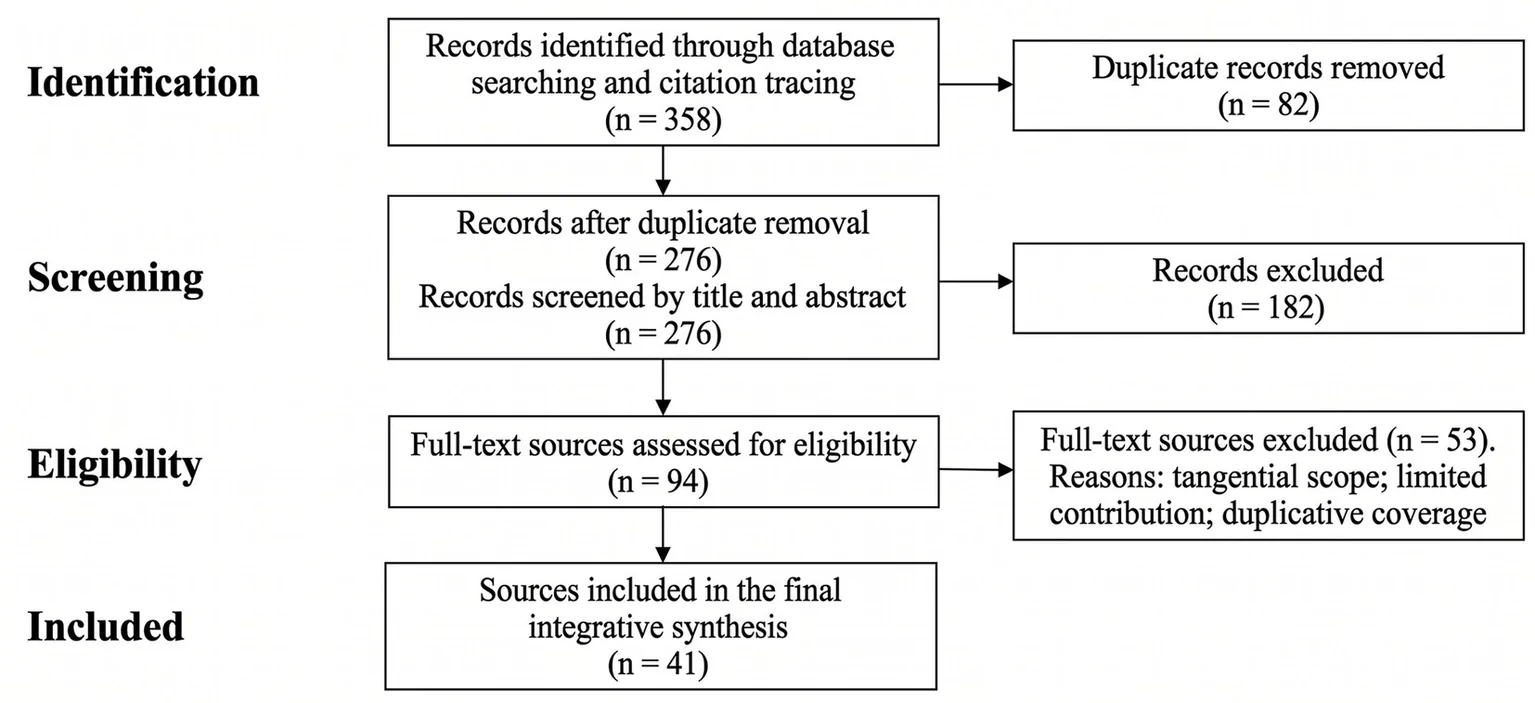
PRISMA-style flow diagram. The figure summarizes record identification, de-duplication, title/abstract screening, full-text eligibility assessment, exclusion, and final inclusion for the structured integrative review.

To increase transparency, the scale of the review process is now reported. The searches and citation tracing identified 358 records. After removing duplicates by title, author, DOI, and publication venue, 276 records remained for title and abstract screening. Of these, 182 were excluded because they were consumer-only algorithm studies, purely technical studies without work-design implications, or publications that did not address autonomy, control, performance, or related psychological mechanisms. Ninety-four full texts were assessed. Fifty-three were excluded at the full-text stage because they were tangential to workplace algorithmic management, lacked a usable conceptual or empirical contribution, or repeated findings already represented by stronger sources. The final retained corpus contained 41 sources, including empirical studies, conceptual and review articles, and selected official legal or policy documents used only for governance claims. The process is visualized in Figure 1.

### Inclusion and exclusion criteria

Sources were included when they met at least one of four criteria: (a) they analyzed algorithmic management or closely related digital control mechanisms in workplace settings; (b) they examined autonomy, empowerment, fairness, stress, resistance, cognitive load, or related mediators relevant to digitally controlled work; (c) they conceptualized or measured performance-related outcomes such as efficiency, quality, work engagement, adaptive performance, counterproductive behavior, exhaustion, or turnover intention; or (d) they provided official legal or policy guidance directly relevant to transparency, human oversight, contestability, worker data protection, or platform governance.

Sources were excluded when they focused only on consumer recommender systems, algorithmic personalization outside work, technical optimization without worker outcomes, speculative commentary without analytical value, or non-work contexts. Non-peer-reviewed sources were generally excluded, except for official legal and policy documents and regulatory enforcement documents that directly informed governance implications.

### Thematic coding and synthesis procedure

The synthesis was thematic rather than statistical because the literature varies strongly in methods, work contexts, construct definitions, and levels of analysis. Coding was conducted in a spreadsheet-based extraction matrix. For each retained source, the following fields were recorded: research context, employment relationship, study method, algorithmic practice, autonomy facet, psychological mediator, outcome category, boundary condition, evidence type, and level of analysis.

Coding proceeded in three rounds. The first round identified recurring algorithmic practices, leading to five practice groups: allocation and nudging, method standardization, surveillance and feedback, rewards and sanctions, and transparency and voice. The second round mapped whether each practice primarily affected scheduling autonomy, method autonomy, decision-making autonomy, or a combination of these facets. The third round mapped mediators and outcomes into psychological states, behavioral performance outcomes, and sustainability outcomes. After initial coding, sources were compared within and across contexts to identify convergent findings, contradictory findings, and claims that rested mainly on theoretical inference.

Because the review was conducted by a single author, formal inter-rater reliability was not calculated. This is a limitation. To minimize subjective bias, the review used a standardized extraction matrix and iterative constant comparison across sources, contexts, methods, and levels of analysis. To reduce opacity, the article reports the screening numbers, coding categories, and synthesis logic in Table 1, uses a PRISMA-style flow diagram to visualize the review process (Figure 1), and includes a comparative evidence table showing how claims differ across contexts and methods. The propositions are therefore presented as testable theoretical propositions rather than as settled empirical conclusions.

**Table 1:** Structured integrative review protocol.

Element	Specification
Review type	Structured integrative review
Databases	Scopus, Web of Science, PsycINFO, ABI/INFORM, ACM Digital Library
Core timeframe	2015 to January 2026, with earlier seminal works added through citation tracing
Search blocks	Algorithmic management/digital monitoring terms + autonomy/mediator terms + performance/outcome terms
Records identified	358
Records after de-duplication	276
Records excluded after title/abstract screening	182
Full texts assessed	94
Full texts excluded	53
Final retained corpus	41
Included evidence	Empirical studies, conceptual and review articles, and selected official policy/legal documents used for governance claims
Main coding categories	Algorithmic practice, autonomy facet, mediator, outcome category, moderator, evidence type, level of analysis
Synthesis logic	Thematic coding followed by cross-context comparison and proposition development
Methodological caution	Single-author coding; standardized extraction matrix and iterative constant comparison used to reduce subjective bias; no statistical meta-analysis; propositions should be tested empirically

## Literature review

### Algorithmic management in the digital workplace

Algorithmic management can be defined as the use of computational systems to perform or structure managerial functions such as allocating tasks, monitoring behavior, evaluating performance, disciplining workers, and shaping access to future work (Lee et al., 2015; Jarrahi et al., 2021). In practice, the phenomenon includes rating systems, scheduling tools, performance dashboards, geolocation tracking, pacing metrics, pricing systems, rewards, deactivation triggers, and rules that constrain human discretion (Kellogg et al., 2020; Stelmaszak et al., 2025).

Early studies concentrated on platform labor, where ride-hailing and delivery platforms organize work through continuous data capture, matching, rating, and pricing systems (Rosenblat and Stark, 2016; Wood et al., 2019; Waldkirch et al., 2021). More recent studies show that algorithmic management also appears in conventional organizations, including freight transport, warehouses, and remote office settings (Bujold et al., 2022; Benlian et al., 2022; Cheon and Erickson, 2025). Employer survey evidence also suggests that these tools are increasingly used for training, monitoring, evaluation, and work organization across countries (Milanez et al., 2025; OECD, 2025).

This broader diffusion matters theoretically. Algorithmic management should not be reduced to one employment model or one technology. It can operate under contractor status, standard employment, or hybrid arrangements. It can be transparent or opaque, developmental or punitive, contestable or unilateral. The review therefore treats algorithmic management as a bundle of software-mediated managerial practices rather than as a homogeneous intervention (Stark and Vanden Broeck, 2024).

### Employee autonomy and autonomy facets

Job autonomy is central to work design theory. In the Job Characteristics Model, autonomy produces experienced responsibility and supports intrinsic motivation (Hackman and Oldham, 1976). Self-Determination Theory similarly treats autonomy as a basic psychological need whose satisfaction supports motivation, well-being, and high-quality functioning (Ryan and Deci, 2000; Gagné and Deci, 2005).

However, autonomy is not unitary. The Work Design Questionnaire distinguishes among work scheduling autonomy, decision-making autonomy, and work methods autonomy (Morgeson and Humphrey, 2006). This distinction is essential in algorithmically managed work because digital systems can enlarge one facet while constraining another. Platform workers may choose when to log in but have limited discretion over pricing, task assignment, or deactivation rules. Warehouse workers may have limited method autonomy under routing and pacing systems. Remote professionals may gain temporal flexibility while losing privacy and discretion under monitoring tools.

This facet-based view is supported by broader work design evidence showing that autonomy effects vary by type and mechanism. Decision-making and method autonomy are especially relevant for motivation, strain reduction, and adaptive performance (Muecke and Greenwald, 2026). Scheduling autonomy can be beneficial, but it may also become ambivalent when flexibility turns into irregularity, overwork, or self-exploitation (Humphrey et al., 2007; Muecke and Iseke, 2019). This review therefore treats autonomy as a profile of discretion over time, technique, and judgment.

### Clarifying performance and performance-related outcomes

A second clarification concerns performance. The term is often used broadly to cover efficiency, work quality, engagement, reliability, effort, and retention. That breadth captures the practical stakes of algorithmic management, but it blurs analytically different outcomes. This review therefore separates three categories.

First, psychological states include work engagement, empowerment, perceived fairness, stress, cognitive load, frustration, and resistance. These states are not performance in the narrow behavioral sense, but they are theoretically proximal mechanisms through which performance effects often unfold (Seibert et al., 2011; Lin et al., 2025).

Second, behavioral performance outcomes include task efficiency, task quality, reliability, adaptive performance, and counterproductive behavior. This distinction follows research showing that individual work performance is multidimensional rather than singular (Koopmans et al., 2011; Koopmans et al., 2014).

Third, sustainability outcomes include sustainable effort, exhaustion, turnover intention, and the viability of continued high performance over time. This category is critical because algorithmic systems may improve short-term speed and compliance while weakening longer-term health, retention, and adaptability. These distinctions are summarized in Table 2 and serve as the conceptual vocabulary for the subsequent analysis.

**Table 2:** Core constructs and analytical distinctions.

Construct	Definition used in this review	Analytical note
Algorithmic management	Software-mediated performance of managerial functions such as allocating, monitoring, evaluating, and sanctioning work	Surveillance is one instrument within algorithmic management, not a synonym for it
Scheduling autonomy	Discretion over when and in what sequence to work	Often expanded in gig and remote work
Method autonomy	Discretion over how tasks are carried out	Often constrained by standardization and routing systems
Decision-making autonomy	Discretion over goals, exceptions, priorities, or problem-solving	Especially important in complex and knowledge work
Psychological states	Engagement, fairness perceptions, stress, cognitive load, frustration, empowerment	Mediators rather than performance itself
Behavioral performance outcomes	Efficiency, quality, reliability, adaptive performance, counterproductive behavior	Performance should not be treated as one undifferentiated outcome
Sustainability outcomes	Sustainable effort, exhaustion, turnover intention, long-term performance viability	Necessary for evaluating whether short-term efficiency gains are durable

### Autonomy under algorithmic control: a differentiated paradox

The main argument is that algorithmic management creates an autonomy paradox, but the paradox is now specified more carefully. What appears contradictory at the general level becomes more intelligible when the analysis asks which autonomy facet is affected, what psychological mechanism is triggered, and which context shapes the outcome. This approach is consistent with broader paradox theory, which treats tensions as persistent and interdependent rather than as contradictions that can be solved by choosing one side (Smith and Lewis, 2011).

Platform workers may experience independence because they can choose when to log in. Yet user ratings, opaque pay calculations, acceptance-rate pressures, and deactivation risks can reduce control over methods, earnings, and future work access (Wood et al., 2019; Cameron, 2024; van Zoonen et al., 2025). Warehouse workers may experience improved coordination and measurable throughput under algorithmic routing, but these gains can be accompanied by reduced method autonomy and intensified pressure (Cheon and Erickson, 2025). Remote professionals may gain time and location flexibility while experiencing monitoring as a loss of trust and privacy, particularly when surveillance is evaluative rather than developmental (Ravid et al., 2023; Siegel et al., 2022; Schlund and Zitek, 2024).

The paradox therefore does not mean that algorithmic management always empowers and constrains workers equally. It means that different design features redistribute autonomy unevenly across time, method, and judgment. This uneven redistribution helps explain why the literature reports divergent findings: one study may capture scheduling flexibility, another may capture method control, and another may capture fairness or stress under monitoring.

### Comparative evidence across contexts, methods, and levels of analysis

A stronger synthesis requires attention to differences in evidence rather than treating all findings as equivalent. The evidence base is heterogeneous. Platform studies often provide rich qualitative accounts of autonomy loss under nominal flexibility, but they sometimes infer performance consequences from worker experience rather than directly measuring performance. Studies in logistics and warehouse settings more directly address monitoring, justice, productivity, or quitting intentions, but their findings may not transfer to knowledge work. Experimental and meta-analytic studies of electronic monitoring provide stronger causal or aggregate evidence for autonomy, stress, satisfaction, and resistance effects, but they do not always capture the full institutional complexity of algorithmic management. Governance documents clarify normative expectations around transparency and oversight, but they are not empirical evidence of performance effects.

The framework should therefore be read as an integrative and mechanism-based synthesis rather than as a claim that all propositions are equally proven. Some relationships are well supported by broader work design theory and monitoring research, such as the link between autonomy-supportive design and motivation. Other claims, especially those connecting algorithmic transparency to long-term sustainable performance, remain plausible but require longitudinal and field-based testing. The propositions are accordingly framed as testable propositions. Table 3 summarizes these differences across evidence streams, typical methods, autonomy insights, outcome implications, and interpretive cautions.

**Table 3:** Evidence comparison across contexts and methods.

Evidence stream	Typical methods	Main autonomy insight	Main outcome insight	Interpretive caution	Evidence stream
Platform and gig work	Qualitative case studies, interviews, surveys, conceptual analysis	Scheduling autonomy can coexist with constrained method and decision autonomy	Performance implications often concern effort, engagement, frustration, and retention rather than direct productivity metrics	Findings may reflect income dependence and platform-specific rules	Platform and gig work
Warehousing, logistics, and transport	Field studies, surveys, HCI studies, employer surveys	Algorithmic pacing and monitoring often reduce method autonomy but may increase coordination	Short-term reliability and throughput may improve; justice and turnover concerns become salient	Routine-task findings may not transfer to creative or professional work	Warehousing, logistics, and transport
Remote and professional monitoring	Experiments, monitoring studies, meta-analyses, organizational surveys	Electronic and algorithmic surveillance can reduce autonomy and increase stress or resistance	Performance effects are mixed and depend on task type and monitoring frame	Monitoring is related to but narrower than algorithmic management	Remote and professional monitoring
Work design and motivation theory	Meta-analyses, validated scales, theoretical models	Method and decision autonomy are central to motivation and adaptation	Autonomy supports engagement and can reduce strain	Evidence is strong but not always algorithmic-specific	Work design and motivation theory
Governance, policy, and legal sources	Official documents, regulatory frameworks, enforcement examples	Transparency, contestability, and human oversight are increasingly treated as necessary safeguards	Governance may reduce legitimacy and turnover risks, but performance evidence remains indirect	Normative guidance should not be mistaken for causal evidence	Governance, policy, and legal sources

### Mechanisms linking perceived autonomy and performance

**Figure 2:**
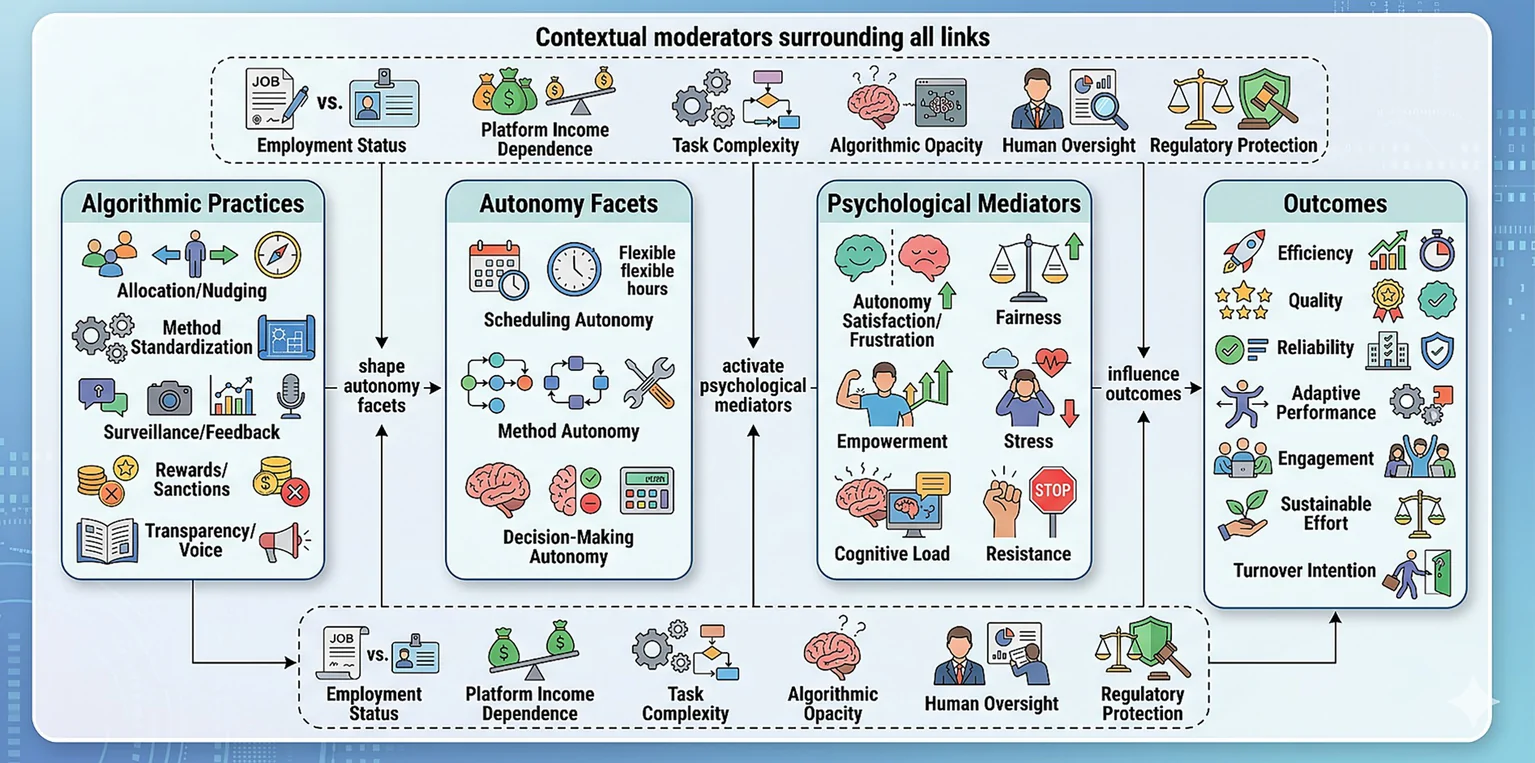
Mechanism-based framework linking algorithmic management practices, autonomy facets, psychological mediators, differentiated outcomes, and boundary conditions.

This section identifies the mechanisms through which algorithmic management is likely to influence perceived autonomy and, through that perception, performance-related outcomes. The mechanisms are presented as differentiated pathways rather than as universal effects (Figure 2).

### Scheduling flexibility versus algorithmic nudging

One commonly advertised advantage of algorithmic management is temporal flexibility. Platform workers may choose working hours, and remote workers may have greater control over location and timing. When workers have genuine alternatives, such flexibility can support work-life integration and increase willingness to work during self-selected periods (Wood et al., 2019).

However, the autonomy value of scheduling flexibility depends on whether choice is genuine or economically compelled. Surge pricing, quest bonuses, dynamic scheduling prompts, and acceptance-rate pressures can convert formal choice into nudge-driven compliance. Cameron’s (2024) analysis is especially useful here: workers may experience frequent choice, but choices remain confined within a strongly structured system. Under high income dependence, workers may feel compelled to follow algorithmic opportunities even when formal scheduling discretion exists. This helps explain why scheduling autonomy can coexist with fatigue, overwork, and frustration (Wood et al., 2019; van Zoonen et al., 2025).

### Method discretion and task execution

Method autonomy concerns how work is performed, not simply when it is performed. This distinction is particularly consequential because algorithmic systems often standardize routes, scripts, picking paths, customer interactions, and workflow steps. In routine, highly measurable tasks, such standardization may improve consistency and reliability. In more complex tasks, however, heavy method control can suppress judgment, adaptation, and local problem solving (Humphrey et al., 2007; Cheon and Erickson, 2025).

The implications are therefore contingent on task complexity. Where output is repetitive and errors are costly, algorithmic method control may improve short-term efficiency. Where work is ambiguous, creative, or relational, method control is more likely to damage adaptive performance and service quality because it prevents workers from applying professional judgment. This claim is supported strongly by general work design theory and plausibly by emerging algorithmic-management evidence, but more direct comparative field studies are needed.

### Surveillance, feedback, and framing

Continuous monitoring is one of the most studied components of digitally managed work. Meta-analytic research shows that electronic monitoring is associated with lower job satisfaction and higher stress, while evidence for performance benefits is limited and context-dependent (Ravid et al., 2023; Siegel et al., 2022). Experimental evidence further suggests that algorithmic surveillance can reduce perceived autonomy and increase resistance more than equivalent human surveillance, especially when monitoring is evaluative rather than developmental (Schlund and Zitek, 2024).

Surveillance therefore affects performance indirectly through psychological interpretation. Workers are more likely to respond negatively when monitoring signals distrust, punishment, or automatic judgment. Developmental framing can reduce this autonomy cost when feedback is informational, improvement-oriented, and separated from automatic discipline. The mechanism is not surveillance per se; it is whether the system is experienced as controlling or autonomy-supportive.

### Rewards, sanctions, and gamification

Algorithmic systems frequently use ratings, leaderboards, badges, acceptance thresholds, and automated rewards or penalties to steer behavior. These mechanisms can increase measurable effort in short-cycle service work where effort is tightly linked to visible targets. However, they also raise concerns about fairness, volatility, and strategic manipulation (Lee, 2018; Cameron, 2024).

The performance implications depend on workers’ interpretations. Reward systems that are predictable, effort-contingent, and correctable may increase motivation and compliance. Systems viewed as opaque, unstable, or unattainable may produce cynicism, gaming, or disengagement. Recent work suggests that algorithmic control can foster engagement through empowerment-like pathways in some gig-work contexts (Lin et al., 2025). That positive pathway is most plausible when control is accompanied by clarity, support, and meaningful work interpretation rather than by constraint alone.

### Transparency voice, and contestability

Transparency and voice are not peripheral governance issues. They shape perceived autonomy because they affect whether workers can understand, contest, and influence the rules that govern them. Research on truck drivers shows that greater transparency in algorithmic surveillance and performance management is associated with stronger perceptions of justice and lower intention to quit, with procedural and distributive justice acting as mediating mechanisms (Bujold et al., 2022). At the same time, emerging evidence warns that transparency is not automatically beneficial. Under some conditions, extensive or poorly designed transparency may increase resistance when workers interpret revealed rules as manipulative or unfair (Hu et al., 2024).

The relevant design principle is therefore not more transparency in the abstract, but actionable transparency. Actionable transparency includes explanation, appeal channels, correctability, and meaningful human oversight. Current European governance developments are relevant here: the Platform Work Directive emphasizes transparency, fairness, human oversight, and accountability in platform work, while the EU AI Act classifies certain employment-related AI systems as high risk (Directive EU, 2024; Regulation EU, 2024).

### Evidence strength and inferential limits

The propositions below are not presented as settled empirical laws. They are integrative statements that combine direct empirical evidence, broader work design theory, and reasonable inference. The strongest evidence concerns the negative psychological effects of controlling surveillance, the importance of job autonomy for motivation and strain, and the role of fairness perceptions in reactions to algorithmic decisions. The evidence is weaker for long-term sustainable performance because many studies are cross-sectional, platform-specific, or focused on proximal psychological states rather than longitudinal performance outcomes.

This distinction matters because algorithmic management research is still developing. The same practice may operate differently across employment relationships, national institutions, task types, and degrees of income dependence. Future research should therefore test the propositions with longitudinal designs, field experiments, and multilevel data that separate worker perceptions from objective or supervisor-rated performance outcomes (Parent-Rocheleau et al., 2024).

### Propositions for future research

Proposition 1. The positive relationship between algorithmic management and worker engagement will be stronger when algorithmic systems preserve or expand method and decision-making autonomy, not merely scheduling autonomy.
Proposition 2. Scheduling autonomy will improve sustainable performance only when algorithmic nudges remain contestable and workers are not highly dependent on a single platform or system for income.
Proposition 3. Evaluative algorithmic surveillance will reduce perceived autonomy and adaptive performance more strongly than developmental algorithmic feedback.
Proposition 4. Transparency, explainability, and appeal rights will improve fairness perceptions and reduce resistance, turnover intention, and disengagement when transparency is actionable rather than symbolic.
Proposition 5. Gamified rewards and algorithmic sanctions will improve short-term efficiency in routine and measurable tasks but will undermine sustainable performance when workers perceive the reward structure as opaque, unstable, or uncorrectable.
Proposition 6. The negative effects of algorithmic autonomy suppression on sustainable performance will be strongest under contractor status, high income dependence, high task ambiguity, high opacity, and weak regulatory protection.

### Boundary conditions

The literature becomes more coherent when examined through boundary conditions rather than broad averages. The same algorithmic practice can produce different outcomes depending on employment status, task complexity, opacity, oversight, income dependence, and regulatory context.

#### Employment status and dependence

Contractor status often amplifies the autonomy paradox because formal independence may coexist with strong unilateral platform control over pricing, rating consequences, and access to work opportunities (Wood et al., 2019; Duggan et al., 2020). High platform income dependence further weakens workers’ ability to refuse algorithmic demands. In standard employment settings, formal protections and managerial escalation channels may partly buffer autonomy costs, although they do not eliminate them.

#### Task complexity

Routine and highly standardized tasks are more compatible with algorithmic specification and pacing. In such settings, behavioral reliability and speed may improve even when autonomy decreases. In complex, relational, or knowledge-intensive tasks, method and decision-making autonomy are more important for quality and adaptiveness. Therefore, the same algorithmic intervention may improve logistics performance yet hinder creative problem-solving or professional judgment.

#### Opacity and human oversight

Opacity intensifies autonomy loss and fairness concerns because workers cannot interpret system decisions or correct errors. Human oversight matters not because it automatically removes control, but because it can restore explanation, discretion, and appeal. This distinction is increasingly recognized in both empirical research and governance frameworks (Bujold et al., 2022; Hu et al., 2024; Directive EU, 2024).

#### Institutional and regulatory context

Legal and institutional context shapes what algorithmic systems can do and how workers experience them. Recent European regulation is especially notable. The Platform Work Directive emphasizes transparency, fairness, human oversight, safety, and accountability in platform algorithmic management. The AI Act recognizes employment-related AI systems for recruitment, task allocation, monitoring, and performance evaluation as high-risk categories requiring safeguards. Regulatory enforcement also illustrates the costs of intrusive monitoring; for example, the French data protection authority fined Amazon France Logistique €32 million for excessive employee monitoring (Commission Nationale de l’informatique et des Libertés, 2024). These developments do not remove the autonomy paradox, but they change its boundary conditions by making opacity and fully automated disciplinary decision-making less institutionally acceptable. Table 4 summarizes how these boundary conditions shift algorithmic management toward more autonomy-supportive or autonomy-suppressive outcome profiles.

**Table 4:** Boundary conditions shaping whether algorithmic management supports or suppresses autonomy.

Boundary condition	More autonomy-supportive pattern	More autonomy-suppressive pattern	Likely outcome profile
Employment relation	Standard employment with protections and escalation channels	Contractor dependence with unilateral platform rules	Greater sustainability in the former; greater precarity in the latter
Task complexity	Routine, measurable, low-discretion tasks	Complex, ambiguous, creative, or relational tasks	Standardization may support reliability in the former but harm adaptation in the latter
Transparency and contestability	Explainable rules, appeal rights, human review, correctability	Opaque metrics, no meaningful recourse	Stronger fairness and lower resistance versus perceived injustice and gaming
Monitoring frame	Developmental and informational feedback	Evaluative and punitive surveillance	Learning and self-regulation versus stress and autonomy frustration
Income dependence	Low to moderate dependence and outside alternatives	High dependence on one platform or system	Genuine flexibility versus coerced compliance
Regulatory context	Clear accountability, worker data rights, oversight requirements	Weak safeguards and limited enforcement	Higher legitimacy and sustainability versus compliance and reputational risks

## Discussion and implications

This review does not claim that the autonomy paradox is a wholly new discovery. Prior studies have already shown that digital workers can experience flexibility and control at the same time. The contribution is more modest and more specific: the review explains how this paradox operates through a three-step logic. Algorithmic practices reallocate different autonomy facets; workers interpret those changes through psychological mechanisms such as fairness, empowerment, stress, cognitive load, and resistance; and outcomes vary according to boundary conditions such as task complexity, income dependence, and institutional protection.

The first implication is conceptual. Autonomy should be measured as a profile rather than as a single score. A system can expand scheduling autonomy while reducing method and decision-making autonomy. Aggregating these facets can hide the most important trade-offs. The second implication concerns performance. Researchers should separate psychological states, behavioral performance, and sustainability outcomes. A system that improves short-term throughput may still damage quality under uncertainty, retention, or sustainable effort. The third implication concerns the boundary between algorithmic management and surveillance. Surveillance is a central mechanism, but algorithmic management also includes allocation, scheduling, reward design, sanctioning, transparency, and voice.

The practical implication is that organizations should not ask whether algorithmic management works in general. They should ask for which tasks, for which workers, through which design features, and over which time horizon it works. In routine and measurable tasks, algorithmic coordination may be justified when accompanied by fairness safeguards and reasonable pacing. In complex, relational, or knowledge-intensive work, method and decision-making autonomy are likely to be more valuable than maximum metric visibility. Human-centered design therefore requires usable explanations, appeal channels, correctability, and human review for consequential decisions. These features are not ethical add-ons; they are part of performance-relevant system design.

The review has limitations. It is not a meta-analysis, and the empirical foundation is heterogeneous across methods, contexts, and constructs. The coding was conducted by a single author, which limits reproducibility. To minimize the potential subjective bias associated with single-author coding, the study used a standardized extraction matrix and iterative constant comparison across sources, contexts, methods, and levels of analysis. Some propositions are grounded in direct empirical findings, whereas others extend broader work design theory to algorithmic contexts and therefore require further testing. Future studies should use longitudinal designs, field experiments, cross-national comparisons, and multilevel data that distinguish scheduling, method, and decision-making autonomy rather than combining them into a single autonomy measure.

## Conclusion

Algorithmic management is neither simply a more efficient form of traditional control nor simply a technologically updated form of domination. Its consequences depend on how it redistributes discretion, how workers interpret that redistribution, and how institutional safeguards shape the employment relationship. The relevant question is therefore not whether algorithmic management creates an autonomy paradox. It often does. The more useful questions are which autonomy facet is expanded or narrowed, through which mechanism, and with what consequences for which type of performance.

Algorithmic systems are more likely to support reliable and sustainable performance when they preserve method and decision-making autonomy, use monitoring as developmental feedback, and provide actionable transparency, contestability, and human review. They are more likely to produce autonomy-poor workplaces when they rely on opaque evaluation, unilateral sanctions, and narrow behavioral programming. Recognizing this distinction is essential for designing digital workplaces that are efficient without eroding the autonomy conditions that make performance sustainable.

## References

[ref1] Benlian A. Wiener M. Cram W. A. Krasnova H. Mädche A. Möhlmann M. . (2022). Algorithmic management: bright and dark sides, practical implications, and research opportunities. Bus. Inf. Syst. Eng. 64, 825–839. doi: 10.1007/s12599-022-00764-w

[ref2] Bujold A. Parent-Rocheleau X. Gaudet M.-C. (2022). Opacity behind the wheel: the relationship between transparency of algorithmic management, justice perception, and intention to quit among truck drivers. Comput. Hum. Behav. Rep. 8:100245. doi: 10.1016/j.chbr.2022.100245

[ref3] Cameron L. D. (2024). The making of the “good bad” job: how algorithmic management manufactures consent through constant and confined choices. Adm. Sci. Q. 69, 458–514. doi: 10.1177/00018392241236163

[ref4] Cheon E. Erickson I. (2025). Fulfillment of the work games: warehouse workers’ experiences with algorithmic management. Proc. ACM Hum. Comput. Interact. 9, 1–30. doi: 10.1145/375740940909183

[ref5] Commission Nationale de l’informatique et des Libertés (2024) Employee Monitoring: CNIL Fined Amazon France Logistique €32 million. Paris, France: Commission Nationale de l’informatique et des Libertés (CNIL).

[ref6] Directive EU (2024). Directive (EU) 2024/2831 of the European Parliament and of the Council of 23 October 2024 on Improving Working Conditions in Platform work. Luxembourg: Official Journal of the European Union.

[ref7] Duggan J. Sherman U. P. Carbery R. McDonnell A. (2020). Algorithmic management and app-work in the gig economy: a research agenda for employment relations and HRM. Hum. Resour. Manag. J. 30, 114–132. doi: 10.1111/1748-8583.12258

[ref8] Gagné M. Deci E. L. (2005). Self-determination theory and work motivation. J. Organ. Behav. 26, 331–362. doi: 10.1002/job.322

[ref9] Hackman J. R. Oldham G. R. (1976). Motivation through the design of work: test of a theory. Organ. Behav. Hum. Perform. 16, 250–279. doi: 10.1016/0030-5073(76)90016-7

[ref10] Hu P. Zeng Y. Wang D. Teng H. (2024). Too much light blinds: the transparency–resistance paradox in algorithmic management. Comput. Hum. Behav. 161:108403. doi: 10.1016/j.chb.2024.108403

[ref11] Humphrey S. E. Nahrgang J. D. Morgeson F. P. (2007). Integrating motivational, social, and contextual work design features: a meta-analytic summary and theoretical extension of the work design literature. J. Appl. Psychol. 92, 1332–1356. doi: 10.1037/0021-9010.92.5.1332, 17845089

[ref12] Jarrahi M. H. Newlands G. Lee M. K. Wolf C. T. Kinder E. Sutherland W. (2021). Algorithmic management in a work context. Big Data Soc. 8, 1–14. doi: 10.1177/20539517211020332

[ref13] Kellogg K. C. Valentine M. A. Christin A. (2020). Algorithms at work: the new contested terrain of control. Acad. Manag. Ann. 14, 366–410. doi: 10.5465/annals.2018.0174

[ref14] Koopmans L. Bernaards C. M. Hildebrandt V. H. de Vet H. C. W. van der Beek A. J. (2014). Measuring individual work performance: identifying and selecting indicators. Work 48, 229–238. doi: 10.3233/WOR-131659, 23803443

[ref15] Koopmans L. Bernaards C. M. Hildebrandt V. H. Schaufeli W. B. de Vet H. C. W. van der Beek A. J. (2011). Conceptual frameworks of individual work performance: a systematic review. J. Occup. Environ. Med. 53, 856–866. doi: 10.1097/JOM.0b013e318226a763, 21775896

[ref16] Lee M. K. (2018). Understanding perception of algorithmic decisions: fairness, trust, and emotion in response to algorithmic management. Big Data Soc. 5, 1–16. doi: 10.1177/2053951718756684

[ref17] Lee M. K. Kusbit D. Metsky E. Dabbish L. (2015) Working with machines: the impact of algorithmic and data-driven management on human workers Proceedings of the 33rd Annual ACM Conference on Human Factors in Computing Systems (CHI ‘15) 1603–1612 New York, NY ACM.

[ref18] Lin Q. Sun R. Zhu Q. (2025). Perceived algorithmic control and gig workers’ work engagement: assessing the mediating role of psychological empowerment and the moderating effect of deep acting. BMC Psychol. 13:1237. doi: 10.1186/s40359-025-03570-7 41204295 PMC12595896

[ref19] Meijerink J. Bondarouk T. (2023). The duality of algorithmic management: toward a research agenda on HRM algorithms, autonomy and value creation. Hum. Resour. Manag. Rev. 33:100876. doi: 10.1016/j.hrmr.2021.100876

[ref20] Milanez A. Lemmens A. Ruggiu C. (2025). Algorithmic management in the workplace: new evidence from an OECD employer survey. OECD Artificial Intelligence Papers No. 31. Paris: OECD Publishing.

[ref21] Morgeson F. P. Humphrey S. E. (2006). The work design questionnaire: developing and validating a comprehensive measure for assessing job design and the nature of work. J. Appl. Psychol. 91, 1321–1339. doi: 10.1037/0021-9010.91.6.1321, 17100487

[ref22] Muecke S. Greenwald J. M. (2026). Linking job autonomy to employee engagement and exhaustion: the role of employee cognitive appraisal. Curr. Psychol. 45:52. doi: 10.1007/s12144-025-08885-7

[ref23] Muecke S. Iseke A. (2019). How does job autonomy influence job performance? A meta-analytic test of theoretical mechanisms. Acad. Manag. Proc. 2019:145. doi: 10.5465/AMBPP.2019.145

[ref24] OECD (2025). How Widespread is Algorithmic Management in Workplaces? Paris, France: OECD Publishing.

[ref25] Parent-Rocheleau X. Parker S. K. Bujold A. Gaudet M.-C. (2024). Creation of the algorithmic management questionnaire: a six-phase scale development process. Hum. Resour. Manag. 63, 25–44. doi: 10.1002/hrm.22185

[ref26] Ravid D. M. White J. C. Tomczak D. L. Miles A. F. Behrend T. S. (2023). A meta-analysis of the effects of electronic performance monitoring on work outcomes. Pers. Psychol. 5–40. doi: 10.1111/peps.12514

[ref27] Regulation EU (2024). Regulation (EU) 2024/1689 of the European Parliament and of the Council of 13 June 2024 Laying down Harmonised rules on artificial Intelligence (Artificial Intelligence Act). Luxembourg: Official Journal of the European Union.

[ref28] Rosenblat A. Stark L. (2016). Algorithmic labor and information asymmetries: a case study of Uber’s drivers. Int. J. Commun. 10, 3758–3784.

[ref29] Ryan R. M. Deci E. L. (2000). Self-determination theory and the facilitation of. Intrinsic motivation, social development, and well-being. Am. Psychol. 55, 68–78. doi: 10.1037/0003-066X.55.1.68, 11392867

[ref30] Schlund R. Zitek E. M. (2024). Algorithmic versus human surveillance leads to lower perceptions of autonomy and increased resistance. Communic. Psychol. 2:53. doi: 10.1038/s44271-024-00102-8, 39242768 PMC11332184

[ref31] Seibert S. E. Wang G. Courtright S. H. (2011). Antecedents and consequences of psychological and team empowerment in organizations: a meta-analytic review. J. Appl. Psychol. 96, 981–1003. doi: 10.1037/a0022676, 21443317

[ref32] Siegel R. König C. J. Lazar V. (2022). The impact of electronic monitoring on employees’ job satisfaction, stress, performance, and counterproductive work behavior: a meta-analysis. Comput. Hum. Behav. Rep. 8:100227. doi: 10.1016/j.chbr.2022.100227

[ref33] Smith W. K. Lewis M. W. (2011). Toward a theory of paradox: a dynamic equilibrium model of organizing. Acad. Manag. Rev. 36, 381–403. doi: 10.5465/amr.2011.59330958

[ref34] Snyder H. (2019). Literature review as a research methodology: an overview and guidelines. J. Bus. Res. 104, 333–339. doi: 10.1016/j.jbusres.2019.07.039

[ref35] Stark D. Vanden Broeck P. (2024). Principles of algorithmic management. Organ. Theory 5:1–24. doi: 10.1177/26317877241257213

[ref36] Stelmaszak M. Möhlmann M. Sørensen C. (2025). When algorithms delegate to humans: exploring human-algorithm interaction at uber. MIS Q. 49, 305–330. doi: 10.25300/MISQ/2024/17911

[ref37] Torraco R. J. (2005). Writing integrative literature reviews: guidelines and examples. Hum. Resour. Dev. Rev. 4, 356–367. doi: 10.1177/1534484305278283

[ref38] van Zoonen W. Treem J. W. Sivunen A. E. (2025). Algorithmic control and work frustration in crowdwork: a case of autonomy suppression and connectivity compulsion. Eur. Manag. J.. Advance online publication. Doi:doi: 10.1016/j.emj.2025.05.001

[ref39] Waldkirch M. Bucher E. Schou P. K. Grünwald E. (2021). Controlled by the algorithm, coached by the crowd: how HRM activities take shape on digital work platforms in the gig economy. Int. J. Hum. Resour. Manag. 32, 2643–2682. doi: 10.1080/09585192.2021.1914129

[ref40] Whittemore R. Knafl K. (2005). The integrative review: updated methodology. J. Adv. Nurs. 52, 546–553. doi: 10.1111/j.1365-2648.2005.03621.x, 16268861

[ref41] Wood A. J. Graham M. Lehdonvirta V. Hjorth I. (2019). Good gig, bad gig: autonomy and algorithmic control in the global gig economy. Work Employ. Soc. 33, 56–75. doi: 10.1177/0950017018785616, 30886460 PMC6380453

